# Supplementary material for: One-directional flow of ionic solutions along fine electrodes under an alternating current electric field
Source: R Soc Open Sci. 2019 Feb 13;6(2):180657. doi: 10.1098/rsos.180657 (PMC6408404; doi:10.1098/rsos.180657)

**One-directional flow of ionic solutions along fine electrodes under an alternating current electric field**

**Figure S1. pH with litmus paper during pumping and after pumping.**


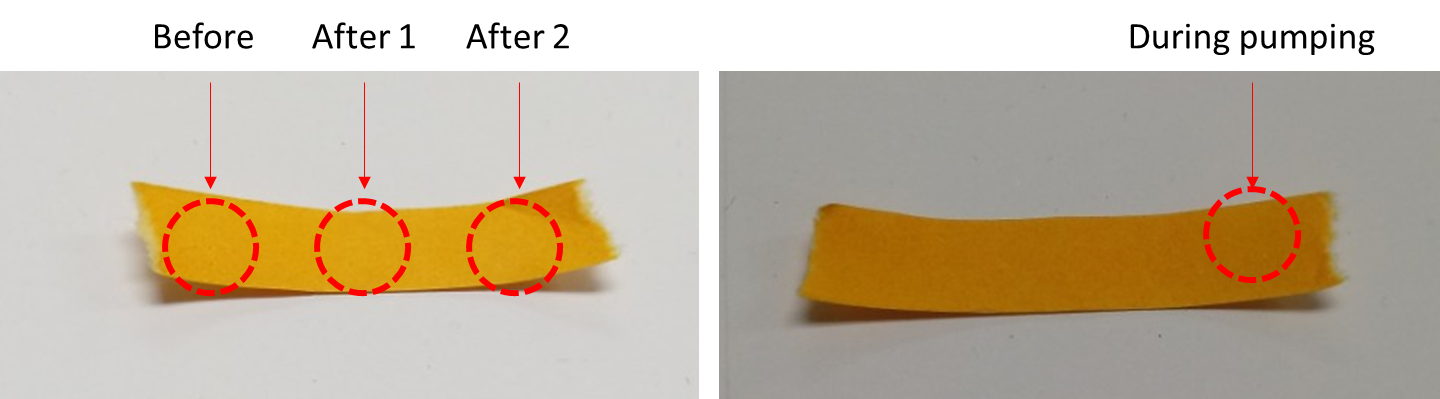


**Figure S2. Flow rates as the applied voltage varied from 5 to 10 V_pp_.**


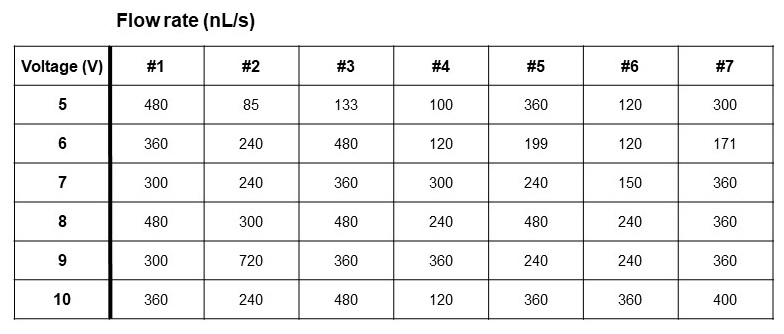


**Figure S3. Flow rates as the applied voltage varied from 5 to 10 V_pp_.**


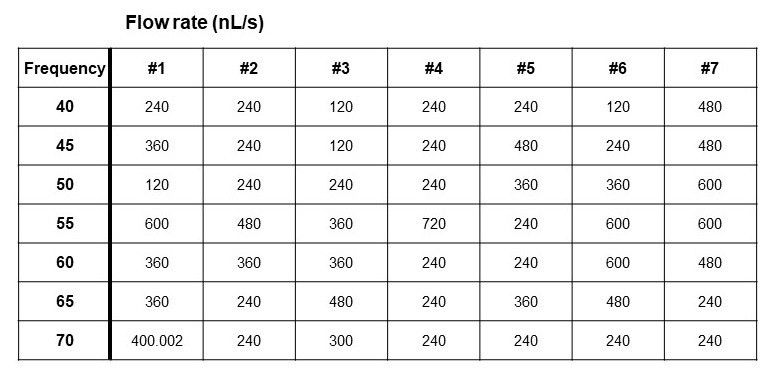


**Figure S4. Flow rates as the off-set voltage varied from −1.0 to 1.0 V.**


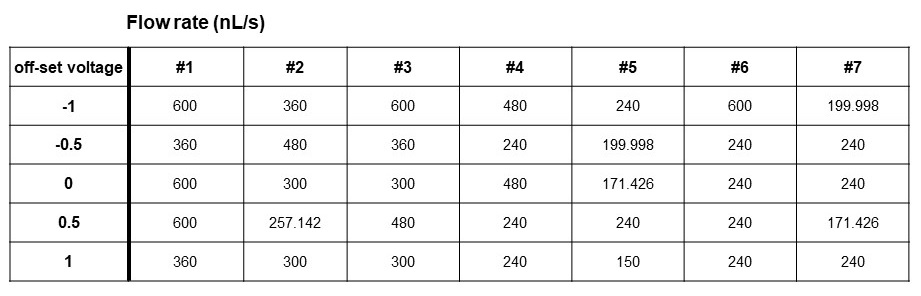


**Figure S5. Flow rates as the ion concentration of the KCl solution varied from 0.01 to 100 mM.**


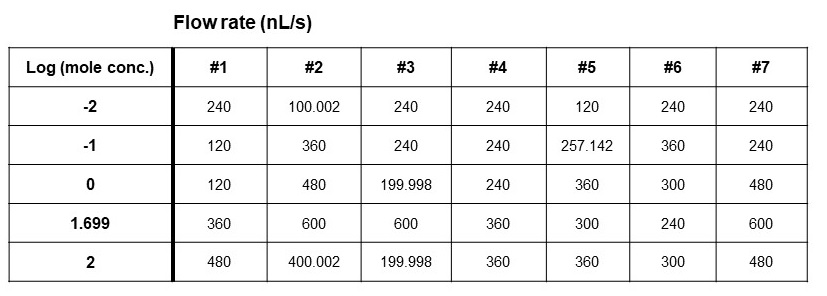


**Figure S6. Pumping frequency ranges generating one-directional flow with CNEs.**


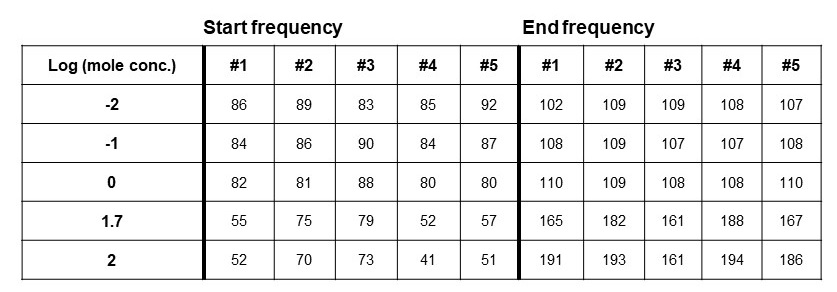

Supplement: Supplementary Figures [file rsos180657supp1.docx]
